# Efficacy, safety and proper dose analysis of PEGylated granulocyte colony-stimulating factor as support for dose-dense adjuvant chemotherapy in node positive Chinese breast cancer patients

**DOI:** 10.18632/oncotarget.18145

**Published:** 2017-05-24

**Authors:** Fan Zhang, RuiXia LingHu, XingYang Zhan, Ruisheng Li, Fan Feng, Xudong Gao, Lei Zhao, Junlan Yang

**Affiliations:** ^1^ Department of Oncology, PLA General Hospital Cancer Center, Institute of Geriatric, PLA General Hospital and Beijing Key Laboratory of Cell Engineering & Antibody, Beijing, China; ^2^ Research Center for Clinical and Translational Medicine, PLA 302 Hospital, Beijing, China; ^3^ Department of Pharmacy, General Hospital of Shenyang Military Command, Shenyang, China; ^4^ Department of Gastroenterology, PLA 302 Hospital, Beijing, China; ^5^ National Clinical Research Center for Normal Aging and Geriatric & The Key Laboratory of Normal Aging and Geriatric, PLA General Hospital and Second Military Medical University, Shanghai, China

**Keywords:** breast cancer, adjuvant dose-dense chemotherapy, neutropenia, PEGylated-recombinant human granulocyte colony stimulating factors

## Abstract

For high-risk breast cancer patients with positive axillary lymph nodes, dose-dense every-two-week epirubicin/cyclophosphamide-paclitaxel (ddEC-P) regimen is the optimal postoperative adjuvant therapy. However, this regimen is limited by the grade 3/4 neutropenia and febrile neutropenia (FN). There is an urgent need to explore the efficacy, safety and proper dosage of PEGylated granulocyte colony-stimulating factor (PEG-G-CSF) as support for ddEC-P in Chinese breast cancer patients with positive axillary lymph nodes. Prospectively, 40 women with stage IIIA to IIIC breast cancer received ddEC-P ± trastuzumab as adjuvant treatment. PEG-G-CSF was injected subcutaneously in a dose of 6 mg or 3 mg on the 2^th^ day of each treatment cycle. With administration of PEG-G-CSF, all of the 40 patients completed 8 cycles of ddEC-P ± trastuzumab regimen without dose reductions or treatment delays. Moreover, no FN cases were observed. Further analysis showed that the proper dosage of PEG-G-CSF was 6 mg for ddEC treatment, and 3 mg for ddP treatment. PEG-G-CSF exhibits advantages compared with G-CSF in convenient of administration and tolerance for high risk Chinese breast cancer patients. More importantly, the proper dose of PEG-G-CSF for high risk Chinese breast cancer patients during ddEC-P chemotherapy may be 6 mg for ddEC treatment and 3 mg for ddP treatment.

## INTRODUCTION

Adjuvant chemotherapy is an effective therapeutic strategy for patients with node positive breast cancer. In order to improve efficacy, various chemotherapy approaches have been employed. Several studies have demonstrated that compared with a conventional three-week schedule, the dose-dense epirubicin plus cyclophosphamide followed by paclitaxel (ddEC-P) regimen can significantly improve disease-free survival (DFS) and overall survival (OS) of the patients with high-risk breast cancer. To achieve improved efficacy, dose-dense adjuvant chemotherapy has been widely used for high risk breast cancer patients [1, 2]. However, the dose-dense regimen requires growth factor support for hematologic recovery between cycles [3, 4]. Thus, it is imperative to prevent myelosuppression and grade 3/4 neutropenia during postoperative dose-dense adjuvant therapy for high-risk breast cancer patients [5, 6]. It is general accepted that administration with myeloid growth factors (mainly including granulocyte colony-stimulating factor, G-CSF) can remit chemotherapy-induced mye-losuppression and facilitate the recovery of marrow functions [7]. However, the short half-life of G-CSF requires daily dosing which limits its wide application in ddEC-P treatment.

Polyethylene glycol (PEG) conjugated G-CSF (PEG-G-CSF), which is characterized by an increased circulating half-life, has the potential to shorten the duration and severity of neutropenia. Moreover, PEG-G-CSF is mainly removed through neutrophil receptor-mediated clearance, contributing to bone marrow reconstitution after each cycle of chemotherapy. Compare with G-CSF, PEG-G-CSF reduces the frequency of injections [8–10]. Burstein and his colleagues revealed that 6mg dose of pegfilgrastim (PEG-G-CSF) per chemotherapy cycle could effectively and safely facilitate every-2-week AC-P (doxorubicin and cyclophosphamide-paclitaxel) regimen. However, the proper dose of pegfilgrastim for HER2 positive patients was not discussed in their study [11]. Among HER2 positive breast cancer, pegfilgrastim is also important for the completion of dose-dense AC (doxorubicin and cyclophosphamide) followed by P/T (paclitaxel/trastuzumab) [12]. All the data of the previous studies showed the extensively application of PEG-G-CSF in dose-dense chemotherapy for various diseases. Given the important role of PEG-G-CSF for high risk breast cancer treatment, there is an urgent need to evaluate the efficacy and proper dose of PEG-G-CSF in adjuvant dose-dense chemotherapy for Chinese node positive HER2+/- breast cancer patients. Here, we evaluated the efficacy, safety and proper dose of PEG-G-CSF in postoperative adjuvant ddEC-P chemotherapy ± trastuzumab among node positive Chinese breast cancer patients, and further explored the proper dosage of PEG-G-CSF during both ddEC treatment and ddP treatment ± trastuzumab regimen.

## RESULTS

### Patient demographics

From June 2014 to January 2015, we enrolled 40 postoperative breast cancer patients with positive axillary lymph nodes (stage IIIA-IIIC) with a median age of 48±16 (38 of them less than 60 years, 2 of them more than 60 years). Among these patients, 34 were premenopausal patients, while 6 patients were postmenopausal. According to the standard of UICC (1997), 5 patients had T1 tumors (T ≤ 2cm), 34 had T2 tumors (2cm < T ≤ 5cm) and 1 had a T3 tumor (> 5cm). Number of metastatic axillary lymph nodes: 3 patients were classified into N1 group (N = 3) while 28 into N2 group (N = 4-9) and 9 into N3 group (N ≥ 10). ER status: 2 patients were ER (+) and the other 38 were ER (-). HER2 status: 12 patients were HER2 (+) while 28 were HER2 (-). Staging: 31 patients were at stage IIIA, while 9 patients were at IIIC stage. Molecular classification: 2 patients were lumina B-like type, 28 patients were triple negative subtype and 10 patients were HER2 positive subtype (Table 1).

**Table 1:** Demographic and characteristics of patients

Characteristic		Case number	%
Age (Year)			
Median	(year)		
Range	48±16 years		
<60 years	31-64 years	38	95%
>=60 years		2	5%
Menopausal status			
Premenopause		34	85%
Postmenopause		6	15%
Tumor size (cm)			
T1 (<2cm)		5	12.5%
T2 (2.1-5cm)		34	85%
T3 (>5cm)		1	2.5%
No. of metastatic axillary lymph nodes			
N1 (3)		3	7.5%
N2 (4-9)		28	70%
N3 (≥10)		9	22.5%
ER status			
+		2	5%
−		38	95%
HER2 status			
+		12	30%
−		28	70%
Staging			
IIIA	31		77.5%
IIIB	0		0
IIIC	9		22.5%
Lumina A-like	0		0
Lumina B-like	2		5%
Triple negative subtype	28		70%
HER2 positive subtype	10		25%

ER: estrogen receptor; HER2: human epidermal growth factor receptor 2.

### Treatment efficacy

**Figure 1:**
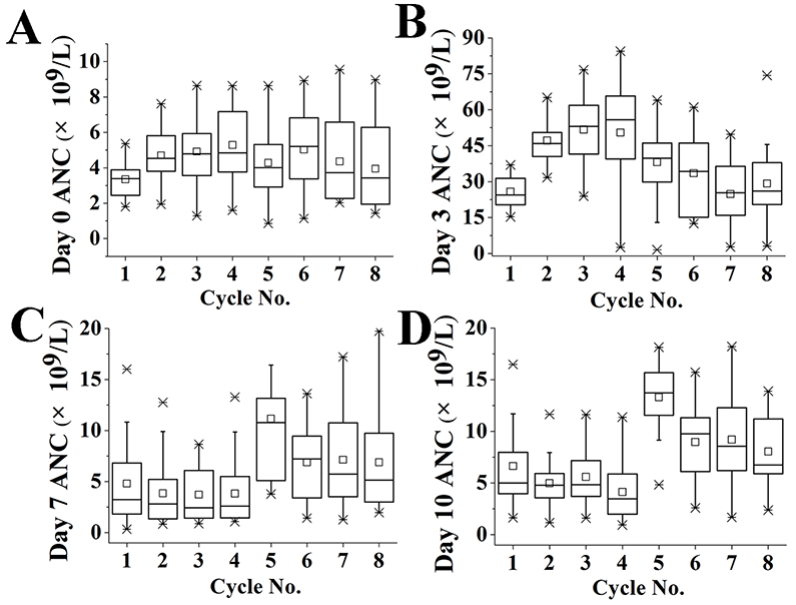
Tendency of absolute neutrophil count after treatment with PEG-G-CSF and ddEC-P chemotherapy Absolute neutrophil counts (ANC) on day0 **(A)**, day3 **(B)**, day7 **(C)** and day10 **(D)** of each treatment cycle are shown. The boxes are drawn from the 25% to 75% quartiles, the horizontal line represents the median, and the whiskers are drawn out to 1.5 × interquartile range (considered extreme values); individual lines represent outliers.

The use of PEG-G-CSF in support of the adjuvant ddEC-P +/- trastuzumab regimen allowed for the successful completion rate of chemotherapy in all patients without dose reductions, treatment delays, or neutropenic fever. The blood counts on the 0th, 3rd, 7th and 10th day of each ddEC-P ± trastuzumab treatment cycle is shown on Table 2 and Figure 1. In ddEC treatment, the overall incidence of neutropenia was 36.25%, and grade3/4 neutropenia occurred in 17.5% (5%) of patients. In ddP treatment, the overall incidence of neutropenia was 15% and grade3/4 neutropenia occurred in 1.25% (0%) of patients (Table 3). In addition, no RT was interrupted due to the therapy toxicity, such as skin, major organs and blood toxicity.

**Table 2:** The average values of ANC of 40 patients tested during every cycle

Cycle	Day	Average value	Cycle	Day	Average value
1	0	3.35	5	0	4.28
	3	25.76		3	38.06
	7	4.8		7	11.16
	10	6.65		10	12.73
2	0	4.7	6	0	5.03
	3	47.14		3	33.5
	7	3.85		7	6.88
	10	4.98		10	8.86
3	0	4.92	7	0	4.36
	3	51.7		3	24.78
	7	3.72		7	7.14
	10	5.59		10	9.02
4	0	5.29	8	0	3.95
	3	50.44		3	29.21
	7	3.82		7	6.89
	10	5.63		10	8.05

ANC: absolute neutrophil count.

**Table 3:** Number and incidence of Grade 1/2 neutropenia, Grade3/4 (Grade4) neutropenia, febrile neutropenia in ddEC treatment

Treatment cycle	Grade 1/2 neutropenia		Grade3/4 (Grade4) neutropenia		FN	
	No.	Incidence (%)	No.	Incidence (%)	No.	Incidence (%)
1-160	58	36.25%	28(8)	17.5%(5%)	0	0
161-320	24	15%	2(0)	1.25%(0)	0	0
Total	82	25.6%	30(8)	9.38%(5%)	0	0

FN: febrile neutropenia; Incidence: cases/numbers of treatment cycle.

(incidence =cases/cycle number)

### The proper dosage of PEG-G-CSF

Among 40 patients, only 1 of them received a starting dose of 3 mg PEG-G-CSF because the weight of this patient was no more than 45kg. The other 39 patients received PEG-G-CSF 6 mg subcutaneous on day 2 of each cycle, according to the NCCN guidelines. In ddEC treatment, 39 patients were treated with 6mg PEG-G-CSF, the incidence of grade 3/4 neutropenia was low. In the subsequent 4 cycles of ddP ± trastuzumab treatment which was adjusted according to the protocol, 20/40 (50%) patients, 26/40 (65%) patients, 38/40 (95%) patients and 40/40 (100%) patients received 3 mg PEG-G-CSF administration respectively, no patients reported grade3/4 neutropenia (Table 4).

**Table 4:** Delivered dosage of PEG-G-CSF

Cycle	6mg of PEG-G-CSF		3mg of PEG-G-CSF	
	Case number	%	Case number	%
1	39	97.5%	1	2.5%
2	39	97.5%	1	2.5%
3	36	90%	4	10%
4	36	90%	4	10%
5	20	50%	20	50%
6	14	35%	26	65%
7	2	5%	38	95%
8	0	0	40	100%

PEG-G-CSF: PEGylated granulocyte colony-stimulating factor.

### Adverse events

With regards to the emergence of bone pain as the most common side effect in patients treated with PEG-G-CSF, we found an incidence of 14 (35%) patients, myalgia 4 (10%), and arthralgia 2 (5.0%). None of the AEs led to the discontinuation of study participation, and none were serious. All of these AEs were easily managed using standard analgesics without any additional treatment. Radiotherapy related adverse events including radiation dermatitis and pneumonitis, all of which were mild or moderate in severity.

## DISCUSSION

Breast cancer is a major cause for cancer-related death among women. Adjuvant chemotherapy has demonstrated efficacy in improving DFS and OS of breast cancer patients, with positive lymph nodes and high risk factors. In order to promote antitumor effects, several approaches are applied to improve the therapeutic effects of conventional 3-week therapeutic regimen. A study carried out by Sparano et al. reported that compared with 3-week paclitaxel schedule, weekly paclitaxel could improve the DFS and OS of breast cancer patients to a certain extent, but its advantages were not significant during the long time follow-up [13]. Until now, the commonly accepted improved therapeutic strategy is dose-dense chemotherapy [14–16]. In line with these findings, a recent systematic review and meta-analysis of adjuvant dose-dense chemotherapy in breast cancer showed that dose-dense chemotherapy could be the preferable adjuvant treatment for either the triple negative or HER2+ subgroup (neither of which express ERs) [17]. Budd et al. demonstrated that dose-dense chemotherapy might be a optimal choice for breast cancer patients with hormone receptor-negative/HER2-negative tumors [3]. Additionally, dose-dense adjuvant chemotherapy has been recommended by NCCN guideline. Therefore, dose-dense regimen was performed for the node positive Chinese breast cancer patients in the current study.

Despite of the improved therapeutic efficacy, myelosuppression-induced neutropenia during dose-dense chemotherapy limits the use and efficacy of this regimen against high-risk breast cancer [18, 19]. G-CSF (filgrastim) with the capacity to increase of ANC and prevention of FN are essential for the completion of dose-dense chemotherapy. Citron et al. reported that hematologic toxicity contributed to 38% of the delays on the conventional 3-week chemotherapy, while, after administration of G-CSF, hematologic toxicity was responsible for only 15% delays in dose-dense therapy [1]. A meta-analysis constructed by Bonilla et al. demonstrated that, with the application of G-CSF, there were no significant differences in the occurrence rate of grade 3 or 4 adverse events between dose-dense and conventional chemotherapy groups [20]. However, the short half-value period of G-CSF can’t significantly prevent FN. It was reported that the occurrence rate of FN was still high (6/11) among patients receiving dose-dense chemotherapy during subsequent hospitalization, despite G-CSF support [23]. PEG-G-CSF may be an ideal alternative to G-SCF in management of dose-dense toxicity. Compared to short-acting G-CSF, the long-acting PEG-G-CSF exhibits advantages not only in preventive and curative effects, but also in side effects, convenience and patients’ living quality. Although PEG-G-CSF is equal to short-acting G-CSF in terms of the incidence rates of fever, debility, and pains in bone, joins and muscle, administration of PEG-G-CSF can markedly reduce the morbidity of FN-induced infection or other complications [9, 21]. Additionally, benefiting from extended half-life, PEG-G-CSF can significantly reduce the times of repetitive injections, improve the quality of life, and reduce the comprehensive costs related to FN therapy [22].

Here, we investigated the protective effect and safety of PEG-G-CSF in Chinese breast cancer patients both with HER2 positive and negative receiving ddEC-P treatment. Analysis results showed that, with the administration of PEG-G-CSF, all of these 40 patients completed 8 cycles of ddEC-P ± trastuzumab regimen without dose reductions or treatment delays. In our study, grade 3/4 neutropenia cases were mainly observed in the period of ddEC regimen. But none of these 40 patients developed to FN during all the chemotherapy treatment cycles. These data indicated that PEG-G-CSF could offer effective bone marrow protection during the ddEC-P treatment in Chinese breast cancer patients.

PEG-G-CSF is an expensive medication, which adds considerable cost to the dose-dense chemotherapy regimen and is associated with adverse events including musculoskeletal pain, fever, chills, body aches, flu symptoms, shortness of breath and allergic reactions [23–27]. In order to reduce economic burden and avoid the toxicities caused by overdose of PEG-G-CSF, we explored the proper dosage of PEG-G-CSF in postoperative ddEC and ddP ± trastuzumab regimen, respectively. As described in NCCN guidelines for breast cancer, the patients’ weights and chemotherapy regimen were two major factors in determining the dosage of PEG-G-CSF. In line with the NCCN guidelines, we found that the recommended dosage of PEG-G-CSF was suitable for the ddEC regimen. However, our results also suggested that 3mg PEG-G-CSF was adequate for Chinese patients with weights no more than 45kg to complete postoperative ddP ± trastuzumab regimen.

Adjuvant radiotherapy (RT) is an effective way to reduce postoperative recurrence and improve clinical outcomes of patients with cancers. However, it was reported that combination with RT and dose-dense chemotherapy in treatment of breast cancer could cause serious side effects, especially pulmonary toxicity [28]. In the present study, RT related adverse events including radiation dermatitis and pneumonitis were observed, but both of these adverse events were mild or moderate in severity that did not discontinue the treatment. A related animal experiment carried out by Kiang et al. reported that PEG-G-CSF could reduce radiation-induced toxicity [29]. Although limitations of PEG-G-CSF was presented for its high cost, the management of PEG-G-CSF might provide protective effects for the breast cancer patients in RT treatment in the current study. However, the long term effects of combination with does-dense chemotherapy and radiotherapy in treatment of Chinese breast cancer patients, as well as the protective value of PEF-G-CSF, will required further investigations.

## CONCLUSION

The use of PEG-G-CSF allows for adequate neutrophil recovery in postoperative Chinese female patients with node positive breast cancer, receiving adjuvant ddEC-P chemotherapy. This study confirms that PEG-G-CSF has an acceptable safety profile in Chinese breast cancer patients. For Chinese patients with body weight more than 45 kg, the proper dose of PEG-G-CSF is 6 mg for ddEC regimen, while 3 mg PEG-G-CSF is an adequate dose to facilitate the administration of ddP +/- trastuzumab.

## MATERIALS AND METHODS

### Patients

This was a prospective study. Forty stage III Chinese female breast cancer patients who were treated at PLA General Hospital were enrolled from June 2014 to January 2015. Eligible patients had positive lymph nodes, regardless of ER, PR, HER2 status or tumor size. Patients were deemed appropriate for dose-dense adjuvant chemotherapy by their oncologists. None of the patients received any prior chemotherapy or radiation therapy. In addition, patients were excluded from the study if they met the following criterion: (1) pregnant or nursing; (2) had a history of hyperviscosity syndrome, bone marrow disorders, such as sickle cell disease, thalassemia or myelodysplasia; (3) immunodeficiency states; (4) previous exposure to G-CSF or erythropoietic agents; (5) concurrent lithium use; (6) RBC transfusion within the previous 4 weeks; (7) antibiotic use within the previous 72 hours; (8) other interconcurrent illness such as active infection, heart failure, or psychiatric illness that might limit treatment compliance. Additionally, patients were required to have normal organ function, defined as follows: absolute neutrophil count (ANC) > 1,500/ L, hemoglobin (Hgb) > 9 mg/dL, platelets > 100,000/L, AST and ALT < 1.5 times the upper limits of normal, left ventricular ejection fraction > 50%, and bilirubin, creatinine, prothrombin time, and partial prothrombin time within institutional normal limits.

### Treatment

Treatment consisted of EC (epirubicin/cyclophosphamide 90/600 mg/m^2^) × 4 intravenously (IV) every 2 weeks followed by P (paclitaxel 175 mg/m^2^) × 4 IV every 2 weeks. Immunohistochemistry (IHC) was applied to evaluate the HER2/neu status of the breast cancer patients. The assay was performed with quick-staining labelled streptavidin-biotin system (LSAB; Dako, USA), followed by diaminobenzidine (DAB) as chromogen. The results of IHC were read in a semiquantitative manner and scored as 0, 1+, 2+, 3+. A score 0 or 1+ was interpretted as negative expression, while a score of 3+ was defined as positive expression for HER2/neu status. A score of 2+ was considered equivocal and fluorescent in situ hybridization (FISH) analysis was performed. The ratio of HER2/neu signal (orange) to centromere 17 (CEP17) signal (green) was used to determine HER-2 amplification. A HER2/CEP17 ratio ≥ 2.0, was read as positive. The FISH result was also confirmed as positive, if the ratio value of HER2/CEP17 < 2.0 and average HER2 copy number ≥ 6.0 [30]. For patients with HER2- positive breast cancer, trastuzumab was started with the first paclitaxel cycle (4 mg/kg bolus followed by 2 mg/kg weekly). At the completion of paclitaxel, trastuzumab was administered every 3 weeks at 6 mg/kg to complete a duration of one year.

Radiation therapy (RT) was given at least 4 weeks after the completion of chemotherapy (chest radiation 50 Gy / 25 fx, medial supraclavicular nodes 45 Gy / 25 fx). The clinical examination was performed once a week for the patients during RT. If more than Grade 2 skin toxicity or other major organ toxicity was observed, RT was interrupted.

### Schedule and dose modifications

Dose reductions of up to 15% were permitted in the next cycle for patient with chemotherapy-related adverse events The grade of the adverse events were defined according to the Common Terminology Criteria for Adverse Events (CTCAE). Patients required adequate hematologic recovery prior to administration of subsequent chemotherapy doses defined by neutrophil and platelet counts greater than or equal to 1.0×10^9^/L and 80×10^9^/L, respectively. If they did not reach those criteria they were allowed an additional 7 days for recovery If the criteria were not met after an additional 7 days, they were given a 15% dose reduction during the same remaining cycles of the same chemotherapy.

### PEG-G-CSF

PEG-G-CSF (purchased from China Shijiazhuang Pharmaceutical Group co., Ltd., CSPC Pharma.) was administered at a dose of 6 mg or 3 mg subcutaneously (SQ) on the 2^th^ day of each treatment cycle, approximately 24 hours after chemotherapy. Complete blood counts were measured on the 0^th^, 3^rd^, 7^th^ and 10^th^ day.

### Dose adjustment

From the perspective of dosage, this study explored the protective effect of PEG-G-CSF, in ddEC-P regimen, on bone marrow through preventive delivery at a level of 6 mg or 3 mg once during the first 4 cycles and the last 4 cycles, respectively. Adjustment protocol for PEG-G-CSF dosage: if the number of neutrophils was more than 5×10^9^/L in the tests on the 3^rd^, 7^th^ and 10^th^ day during one certain cycle as well as on the 0^th^ day of the next cycle, PEG-G-CSF was given at a level of 3 mg after the next cycle of treatment; when the number of neutrophils was less than 5×10^9^/L in the monitoring on the same four time points after the application of 3mg, the dosage of PEG-G-CSF would be returned to 6mg dose for subsequent cycles.

### Objectives

Primary endpoints were: 1) The completion rates of treatment; 2) Incidence of dose reduction and treatment delays of ddEC-P regimen.

Secondary endpoints were: 1) Incidence of FN: FN was defined as a single temperature ≥38.3°C or ≥38.0°C sustained over 1 h accompanied by an absolute neutrophil count (ANC) < 1000 k/μL (with the expectation of further decline to <500 k/μL) [31]; 2) Incidence of grade 3/4 neutropenia; 3) Proper dosage of PEG-G-CSF.

## References

[1] Citron ML, Berry DA, Cirrincione C, Hudis C, Winer EP, Gradishar WJ, Davidson NE, Martino S, Livingston R, Ingle JN, Perez EA, Carpenter J, Hurd D (2003). Randomized trial of dose-dense versus conventionally scheduled and sequential versus concurrent combination chemotherapy as postoperative adjuvant treatment of node-positive primary breast cancer: first report of Intergroup Trial C9741/Cancer and Leukemia Group B Trial 9741. J Clin Oncol.

[2] Buzdar AU (2007). Adjuvant chemotherapy for high-risk operable breast cancer. J Clin Oncol.

[3] Budd GT, Barlow WE, Moore HC, Hobday TJ, Stewart JA, Isaacs C, Salim M, Cho JK, Rinn KJ, Albain KS, Chew HK, Burton GV, Moore TD (2015). SWOG S0221: a phase III trial comparing chemotherapy schedules in high-risk early-stage breast cancer. J Clin Oncol.

[4] Burnell M, Levine MN, Chapman JA, Bramwell V, Gelmon K, Walley B, Vandenberg T, Chalchal H, Albain KS, Perez EA, Rugo H, Pritchard K, O’Brien P, Shepherd LE (2010). Cyclophosphamide, epirubicin, and fluorouracil versus dose-dense epirubicin and cyclophosphamide followed by paclitaxel versus doxorubicin and cyclophosphamide followed by paclitaxel in node-positive or high-risk node-negative breast cancer. J Clin Oncol.

[5] Kuderer NM, Wolff AC (2014). Enhancing therapeutic decision making when options abound: toxicities matter. J Clin Oncol.

[6] Weycker D, Barron R, Edelsberg J, Kartashov A, Lyman GH (2012). Incidence of reduced chemotherapy relative dose intensity among women with early stage breast cancer in US clinical practice. Breast Cancer Res Treat.

[7] Falandry C, Campone M, Cartron G, Guerin D, Freyer G (2010). Trends in G-CSF use in 990 patients after EORTC and ASCO guidelines. Eur J Cancer.

[8] Vogel CL, Wojtukiewicz MZ, Carroll RR, Tjulandin SA, Barajas-Figueroa LJ, Wiens BL, Neumann TA, Schwartzberg LS (2005). First and subsequent cycle use of pegfilgrastim prevents febrile neutropenia in patients with breast cancer: a multicenter, double-blind, placebo-controlled phase III study. J Clin Oncol.

[9] Rossi L, Tomao F, Lo Russo G, Papa A, Zoratto F, Marzano R, Basso E, Giordani E, Verrico M, Ricci F, Pasciuti G, Francini E, Tomao S (2013). Efficacy and safety analysis of once per cycle pegfilgrastim and daily lenograstim in patients with breast cancer receiving adjuvant myelosuppressive chemotherapy FEC 100: a pilot study. Ther Clin Risk Manag.

[10] Bondarenko I, Gladkov OA, Elsaesser R, Buchner A, Bias P (2013). Efficacy and safety of lipegfilgrastim versus pegfilgrastim: a randomized, multicenter, active-control phase 3 trial in patients with breast cancer receiving doxorubicin/docetaxel chemotherapy. BMC Cancer.

[11] Burstein HJ, Parker LM, Keshaviah A, Doherty J, Partridge AH, Schapira L, Ryan PD, Younger J, Harris LN, Moy B, Come SE, Schumer ST, Bunnell CA (2005). Efficacy of pegfilgrastim and darbepoetin alfa as hematopoietic support for dose-dense every-2-week adjuvant breast cancer chemotherapy. J Clin Oncol.

[12] Dang C, Fornier M, Sugarman S, Troso-Sandoval T, Lake D, D’Andrea G, Seidman A, Sklarin N, Dickler M, Currie V, Gilewski T (2008). The safety of dose-dense doxorubicin and cyclophosphamide followed by paclitaxel with trastuzumab in HER-2/neu overexpressed/amplified breast cancer. J Clin Oncol.

[13] Sparano JA, Zhao F, Martino S, Ligibel JA, Perez EA, Saphner T, Wolff AC, Sledge GW, Wood WC, Davidson NE (2015). Long-term follow-up of the E1199 phase III trial evaluating the role of taxane and schedule in operable breast cancer. J Clin Oncol.

[14] Mayer EL, Burstein HJ (2008). Weighing a dose-dense option for adjuvant chemotherapy and trastuzumab in early-stage breast cancer. J Clin Oncol.

[15] Fornier M, Norton L (2005). Dose-dense adjuvant chemotherapy for primary breast cancer. Breast Cancer Res.

[16] Swain SM, Tang G, Geyer CE, Rastogi P, Atkins JN, Donnellan PP, Fehrenbacher L, Azar CA, Robidoux A, Polikoff JA, Brufsky AM, Biggs DD, Levine EA (2013). Definitive results of a phase III adjuvant trial comparing three chemotherapy regimens in women with operable, node-positive breast cancer: the NSABP B-38 trial. J Clin Oncol.

[17] Petrelli F, Cabiddu M, Coinu A, Borgonovo K, Ghilardi M, Lonati V, Barni S (2015). Adjuvant dose-dense chemotherapy in breast cancer: a systematic review and meta-analysis of randomized trials. Breast Cancer Res Treat.

[18] Barcenas CH, Niu J, Zhang N, Zhang Y, Buchholz TA, Elting LS, Hortobagyi GN, Smith BD, Giordano SH (2014). Risk of hospitalization according to chemotherapy regimen in early-stage breast cancer. J Clin Oncol.

[19] Zauderer M, Patil S, Hurria A (2009). Feasibility and toxicity of dose-dense adjuvant chemotherapy in older women with breast cancer. Breast Cancer Res Treat.

[20] Bonilla L, Ben-Aharon I, Vidal L, Gafter-Gvili A, Leibovici L, Stemmer SM (2010). Dose-dense chemotherapy in nonmetastatic breast cancer: a systematic review and meta-analysis of randomized controlled trials. J Natl Cancer Inst.

[21] Pietri E, Andreis D, Fabbri F, Menna C, Schirone A, Kopf B, Rocca A, Amadori D, De Giorgi U (2015). A phase II study of a dose-density regimen with fluorouracil, epirubicin, and cyclophosphamide on days 1 and 4 every 14 days with filgrastim support followed by weekly paclitaxel in women with primary breast cancer. Oncologist.

[22] Holmes FA, Jones SE, O’Shaughnessy J, Vukelja S, George T, Savin M, Richards D, Glaspy J, Meza L, Cohen G, Dhami M, Budman DR, Hackett J (2002). Comparable efficacy and safety profiles of once-per-cycle pegfilgrastim and daily injection filgrastim in chemotherapy-induced neutropenia: a multicenter dose-finding study in women with breast cancer. Ann Oncol.

[23] Potosky AL, Malin JL, Kim B, Chrischilles EA, Makgoeng SB, Howlader N, Weeks JC (2011). Use of colony-stimulating factors with chemotherapy: opportunities for cost savings and improved outcomes. J Natl Cancer Inst.

[24] Aarts MJ, Grutters JP, Peters FP, Mandigers CM, Dercksen MW, Stouthard JM, Nortier HJ, van Laarhoven HW, van Warmerdam LJ, van de Wouw AJ, Jacobs EM, Mattijssen V, van der Rijt CC, Smilde TJ (2013). Cost effectiveness of primary pegfilgrastim prophylaxis in patients with breast cancer at risk of febrile neutropenia. J Clin Oncol.

[25] Hershman DL, Wilde ET, Wright JD, Buono DL, Kalinsky K, Malin JL, Neugut AI (2012). Uptake and economic impact of first-cycle colony-stimulating factor use during adjuvant treatment of breast cancer. J Clin Oncol.

[26] Montella L, Addeo R, Guarrasi R, Cennamo G, Faiola V, Capasso E, Caraglia M, Del Prete S (2010). Once-per-cycle pegfilgrastim in breast cancer patients treated with docetaxel/epidoxorubicin/cyclophosphamide. Eur J Cancer Care (Engl).

[27] Kirshner JJ, Heckler CE, Janelsins MC, Dakhil SR, Hopkins JO, Coles C, Morrow GR (2012). Prevention of pegfilgrastim-induced bone pain: a phase III double-blind placebo-controlled randomized clinical trial of the university of rochester cancer center clinical community oncology program research base. J Clin Oncol.

[28] Todd NW, Peters WP, Ost AH, Roggli VL, Piantadosi CA (1993). Pulmonary drug toxicity in patients with primary breast cancer treated with high-dose combination chemotherapy and autologous bone marrow transplantation. Am Rev Respir Dis.

[29] Kiang JG, Zhai M, Liao PJ, Bolduc DL, Elliott TB, Gorbunov NV (2014). Pegylated G-CSF inhibits blood cell depletion, increases platelets, blocks splenomegaly, and improves survival after whole-body ionizing irradiation but not after irradiation combined with burn. Oxid Med Cell Longev.

[30] Wolff AC, Hammond ME, Hicks DG, Dowsett M, McShane LM, Allison KH, Allred DC, Bartlett JM, Bilous M, Fitzgibbons P, Hanna W, Jenkins RB, Mangu PB (2013). Recommendations for human epidermal growth factor receptor 2 testing in breast cancer: American Society of Clinical Oncology/College of American Pathologists clinical practice guideline update. J Clin Oncol.

[31] Butts AR, Bachmeier CC, Dressler EV, Liu M, Cowden A, Talbert J, Adams VR (2017). Association of time to antibiotics and clinical outcomes in adult hematologic malignancy patients with febrile neutropenia. J Oncol Pharm Pract.

